# Literary Exchanges

**Published:** 1868-12-15

**Authors:** 


LITERARY EXCHANGES.—The *Atlantic*, *Our Young Folks*, and *Every Saturday*, published by Fields, Osgood & Co., (successors to Ticknor & Fields) show marked improvement under the new *regime.* We cordially commend each to the patronage of the families of our readers.——The New York *Independent* comes to us enlarged to mammoth proportions. It is probably the largest single sheet periodical now issued in the world. To a man who wishes to keep "posted" in what the world is thinking about—irrespective of political or theological opinions—it affords "just the thing." It is *Independent* to the verge of audacity, and, although mammoth in its proportions, its lucubrations are never heavy or unreadable. The price remains the same as before the enlargement.——Our thanks are due to Messrs. Turnbull & Murdoch, of Baltimore, for a proffered exchange with the *New Eclectic Magazine*, published by them. The *New Eclectic* commences its fourth volume on January, 1869. It is handsomely gotten up, and is filled with excellent and spicy reading matter. The January number contains a fine portrait of John Ruskin, the great Art critic. $4.00 per annum. Handsome discount to clubs.

